# Validating the Iowa Test of Consonant Perception in a large cohort of cochlear implant users

**DOI:** 10.1121/10.0035804

**Published:** 2025-02-10

**Authors:** Francis X. Smith, Joel I. Berger, Phillip E. Gander, Adam T. Schwalje, Timothy D. Griffiths, Bob McMurray, Inyong Choi

**Affiliations:** 1 Department of Otolaryngology—Head and Neck Surgery, University of Iowa Hospitals and Clinics, Iowa City, Iowa 52242, USA; 2 Department of Neurosurgery, University of Iowa Hospitals and Clinics, Iowa City, Iowa 52242, USA; 3 Otolaryngology Clinics, Unity Point Health, Moline, Illinois 61265, USA; 4 Biosciences Institute, Newcastle University, Newcastle upon Tyne NE2 4HH, United Kingdom; 5 Department of Psychological and Brain Sciences, University of Iowa, Iowa City, Iowa 52242, USA; 6 Department of Communication Sciences and Disorders, University of Iowa, Iowa City, Iowa 52242, USA; 7 Department of Intelligence and Information, Seoul National University, Seoul, South Korea francis-smith@uiowa.edu, joel-berger@uiowa.edu, phillip-gander@uiowa.edu, adam.schwalje@gmail.com, tim.griffiths@newcastle.ac.uk, bob-mcmurray@uiowa.edu, inyong-choi@uiowa.edu

## Abstract

The Iowa Test of Consonant Perception (ITCP) was designed to test word-initial phoneme perception by uniformly sampling frequently used phonemes as well as balancing feature overlap of response competitors. However, the task has only been validated in normal hearing listeners. In this study, a large cohort of cochlear implant users completed the ITCP and two commonly used clinical measures of speech recognition [AzBio sentences and consonant-nucleus-consonant (CNC) words]. At two different signal-to-noise ratios, the ITCP showed strong convergent validity with other speech recognition tasks and good test-retest reliability. The ITCP is a useful tool for both clinicians and experimental researchers.

## 1. Introduction

Improving speech intelligibility is a critical outcome of hearing interventions, like cochlear implants (CIs). However, the substantial variability in speech intelligibility between listeners after the intervention demands accurate and efficient methods of assessment to optimize device tuning to the listener's need and to have an accurate understanding of how people are likely to experience the intervention. Many types of tests are used to assess different aspects of speech intelligibility in CI users. Two speech recognition assessments widely used for CI users are the AzBio sentence intelligibility test (Spahr *et al.*, 2012) and the consonant-nucleus-consonant (CNC) word recognition test (Luxford, 2001). Both tests are open set tasks in which a participant is asked to repeat a sentence or word.

Open set tasks have gained popularity as they offer a good picture of overall speech recognition ability and may resemble the demands of real-world language processing closely. Critically, as chance performance is near zero in open set tasks, they are less susceptible to ceiling effects that may limit the diagnostic ability of the test at higher levels of performance. However, open set tasks have several challenges. Successful performance on the task also requires lexical retrieval, decision making, and speech production. Consequently, scores may reflect more than an individual's ability to correctly perceive speech, particularly in the context of older adults or people with variable cognitive or speech-motor abilities. Moreover, open set tasks must be scored manually and thus are generally reported only with overall accuracy scores. In contrast, while closed set tasks are not as common, they can allow for automated scoring and item-level analysis (Owens and Schubert, 1977), while minimizing decision and speech production demands.

Similarly, sentence tasks like AzBio are more ecologically valid. However, as tests of hearing ability, they may be less precise, as variation in performance will be in part a product of variation in things like language skill, vocabulary, working memory, and speech production. These are skills that are important for real-world function, but quite out of the immediate influence of interventions that restore or improve access to auditory input. Thus, single-word tests (particularly if they are well balanced across the sounds of the language) may offer an important complement that can isolate hearing ability.

In that vein, the Iowa Test of Consonant Perception (ITCP) (Geller *et al.*, 2020) was developed as a new task to assess single-word recognition using a closed set, four-alternative forced-choice task. This type of task removes the need for the participant to produce speech in order to respond, removing speech production errors as a confounding source of variability in performance. The ITCP is implemented as a relatively quick task that can be completed by patients autonomously, which may be useful for both researchers and clinicians. Because the ITCP broadly, and relatively evenly, samples the phonetic space, it has diagnostic strength when evaluating speech recognition in specific contexts. For example, the ITCP can be used to quickly identify if a particular phoneme is difficult for a listener as well as assess what types of errors occur most frequently. This could inform device tuning at a more fine-grained level than is available with coarse accuracy measures.

The ITCP was validated using normal hearing (NH) listeners who were tested remotely using online presentation of stimuli via headphones (Geller *et al.*, 2021). Participants performed the ITCP task, the CNC word task, and the AzBio sentence task. Despite the considerable difference in task demands, the ITCP showed good convergent validity with both CNC and AzBio scores. This suggests that the ITCP is tapping into similar speech recognition abilities between both the single-word and sentence tasks. The ITCP was shown to have good test-retest reliability in NH listeners as well. The reliability and validity of the ITCP as a measure of speech recognition suggest that it could be useful for clinical purposes.

However, the ITCP has not yet been evaluated for use as clinical speech recognition assessment with CI users. It is important to verify that it still has test-retest reliability within a clinical population, as well as convergent validity with other speech recognition measures. While the ITCP was previously validated in NH listeners listening to stimuli via headphones, the current study evaluates CI patients with stimuli presented in a sound field via a loudspeaker. If certain phonemes differ in intelligibility for CI users compared to NH listeners, the ITCP may show different test-retest reliability or convergent validity. Speech perception tasks are an important measure to assess CI patient outcomes over time after implantation, and good test-retest reliability is critical for accurately assessing these changes. Additionally, this study provides data on the distribution of scores on the ITCP at two different noise levels that may serve as a benchmark for comparison with future data from CI patients. In the present study, we measure a large cohort of CI users' performance on the ITCP, as well as their performance on the CNC and AzBio tasks as administered by their audiologists during their annual visit for device tuning. We assess convergent validity using CNC and AzBio in nearly all participants, as well as test-retest reliability among a further subset of participants who returned for an additional visit.

## 2. Methods

### 2.1 Participants

One hundred twenty experienced CI patients (with more than one year of device experience), between 20 and 83 years of age [mean (*M*) = 63.8 years, standard deviation (SD) = 12.3 years; 62 female], were recruited for this study. Participants were recruited through a patient registry maintained by the University of Iowa Department of Otolaryngology, and most were tested during their annual clinical visit for audiological examination and device tuning. Participants were required to be over 18 years of age, have no known neurological disorders, and not have single-sided deafness. Both pre- and post-lingually deafened participants were included in this study. A full breakdown of the patients' device configuration/type, demographic, and audiological factors can be found in the supplementary material. Out of these 120 participants, 44 were tested on the ITCP during an additional visit to assess test-retest reliability. These visits ranged from one month to three years apart from their initial test date (*M* = 13.2 months, SD = 7.7 months). Patients provided written consent to participate in the ITCP, and all study procedures were approved by the University of Iowa Institutional Review Board.

This sample size was chosen on the basis of convenience (we tested all patients who came through the lab in a fixed period), not power. A *post hoc* sensitivity analysis (assuming α = 0.05, 1-β = 0.80) found a minimum detectable effect of |*r*| > 0.249 for the full sample of 120 and |*r*| > 0.389 for the test/retest sample—effects far lower than we expected given our prior work.

### 2.2 Materials and procedures

All participants performed the ITCP during a research session that included several other tasks. The ITCP was always the first task. In a subset of CI users, we obtained performance on two common clinical tests of speech recognition: CNC word recognition (in quiet; *n* = 111) and AzBio sentence recognition [in noise, +10 dB signal-to-noise ratio (SNR) from the front; *n* = 84]. These were administered by a trained audiologist in a separate clinical session as part of a routine audiological examination. If the CNC and AzBio tests were administered on a different day than the ITCP, we accepted tests performed within ±90 days of the ITCP testing date. Participants were excluded from analyses if we did not have complete data for all measures included in that analysis. All tasks were performed in a sound field with the individual CI user's common listening configuration to replicate their daily listening conditions. The presentation level was 70 dB SPL.

### 2.3 ITCP

The ITCP was implemented using matlab (Mathworks, 2020) scripts utilizing psychtoolbox 3 (Brainard, 1997; Pelli, 1997). The experiment was conducted in an acoustically treated booth using a single loudspeaker (JBL model LOFT40; Harmon International, Stamford, CT) positioned at 0° azimuth at a distance of 1.2 m. Visual stimuli were presented via a computer monitor located 0.5 m in front of the participant at eye level. Sound levels were the same across participants and were calibrated to present two distinct SNRs, as described later.

Each trial began with a white fixation cross appearing on a black background. The fixation cross remained on the screen throughout the presentation of auditory stimuli. After 500 ms, multi-talker babble began. The babble always persisted for 2000 ms, and a target word would onset after 1000 ms of the babble had played. One hundred millseconds after the multi-talker babble offset, four words appeared at the center of the screen, with one word per line (labeled on the left side of the word with numbers 1 through 4), and participants were instructed to choose the word that they heard during the trial, using a keypad to select the number corresponding to one of the four options. For example, if a participant heard the word “lip” they would see response options “1: zip 2: lip 3: yip 4: rip,” with each option displayed on a separate line of text.

The ITCP consists of 120 target words organized into 30 item sets, in which a given target and its three foils differ only in their initial consonant. [For full details on the development of the ITCP item sets, see Geller *et al.* (2021).] Each of these target words occurred under both a high-SNR condition (+15 dB) and low-SNR condition (+7.5 dB) with eight-talker multi-talker babble. These SNR levels were chosen based on both pilot data and previous work with CI patients in another closed set task (Berger *et al.*, 2023). The low-SNR condition was intended to produce a challenging listening experience that avoided both floor and ceiling level performance, while the high-SNR condition was intended to produce an easy condition that still included a low level of background noise. The noise level was manipulated to create the two SNR conditions while the target word presentation level remained constant. Multi-talker babble was chosen in line with the original ITCP validation with NH listeners due to its ecological validity. Eight-talker babble was used to decrease the potential for informational masking of target words by speech segments included in the babble compared to multi-talker babble with fewer talkers. Two distinct talkers (one male, one female) were used for this test, and each speaker was used for all 120 target words. A given talker's production of a target was randomly assigned to one of the two noise conditions, and this assignment was counterbalanced across participants. This led to 240 trials in total per participant, with 120 trials under the high-SNR condition and 120 trials under the low-SNR condition. Conditions were randomly interleaved, and the order of trials was randomized for each participant. The task took approximately 15 min after setup and instruction.

## 3. Results

We begin by reporting descriptive statistics for accuracy on the ITCP as well as accuracy for CNC and AzBio tasks. We then assess the convergent validity of the ITCP with correlational analyses between the ITCP, CNC, and AzBio scores as well as assessing the test-retest reliability for a subset of our participants.

Under the high-SNR condition, the participants' mean proportion correct had a range of 0.31–0.97 (*M* = 0.71, SD = 0.14). Under the low-SNR condition, the participants' mean proportion correct had a range of 0.23–0.90 (*M* = 0.57, SD = 0.13). The means of the high- and low-SNR conditions for the ITCP were significantly different [*t*(119) = 25.4, *p* < 0.001], confirming that the noise manipulation impacted performance on the task. In the CNC word task in quiet, the participants' mean proportion correct had a range of 0.16–1.00 (*M* = 0.76, SD = 0.17). In the AzBio sentence task in noise, the participants' mean proportion ranged from 0.00 to 1.00 (*M* = 0.46, SD = 0.28).

### 3.1 Convergent validity

**Fig. 1.**
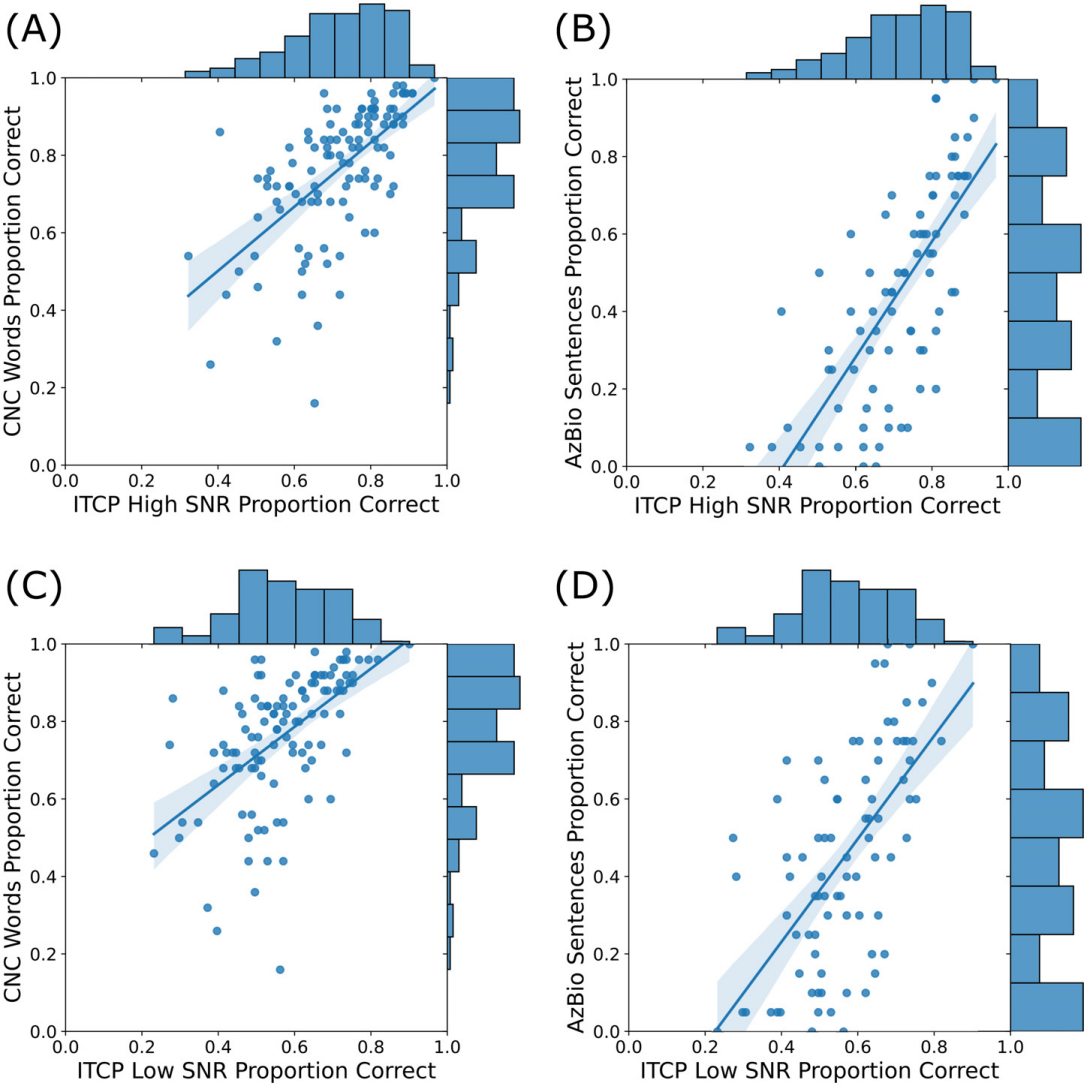
Scatterplots and histograms of the ITCP (high- and low-SNR conditions), CNC, and AzBio scores. (A) Scatterplot of high-SNR ITCP and CNC word scores along with distribution of data (histograms). (B) Scatterplot of high-SNR ITCP and AzBio sentences scores along with distribution of data (histograms). (C) Scatterplot of low-SNR ITCP and CNC word scores along with distribution of data (histograms). (D) Scatterplot of low-SNR ITCP and AzBio sentence scores along with distribution of data (histograms).

We assess convergent validity of the ITCP in our CI population by correlating performance under both the high- and low-SNR conditions of the ITCP with performance on CNC words (in quiet) and AzBio sentences (in noise, +10 dB SNR from the front). The Pearson correlation coefficient was used for all validity comparisons. We found a significant positive correlation between ITCP performance under this high-SNR condition and CNC word scores [*r* = 0.63, *t*(109) = 8.48, *p* < 0.001] [Fig. 1(A)]. We also found a significant positive correlation between ITCP performance under the high-SNR condition and AzBio sentence scores [*r* = 0.73, *t*(82) = 9.65, *p* < 0.001] [Fig. 1(B)]. Correlations between ITCP performance under the low-SNR condition were somewhat smaller (though still large in absolute terms), but performance still showed significant positive correlations with both CNC word scores [*r* = 0.55, *t*(109) = 7.04, *p* < 0.001] [Fig. 1(C)] and AzBio sentence scores [*r* = 0.64, *t*(82) = 7.47, *p* < 0.001] [Fig. 1(D)]. The correlation between CNC word scores and AzBio sentence scores was also significant [*r* = 0.77, *t*(82) = 10.85, *p* < 0.001] (figure not shown for brevity). A Spearman rank-order correlation showed strong monotonicity in the relationship between ITCP scores under the high- and low-SNR conditions [*r*_s_(118) = 0.91, *p* < 0.001].

Given the wide age range of the participants included in the sample, we also ran a series of linear regressions that included the age of the participant as a covariate to confirm that the relationship between AzBio/CNC scores and the ITCP (both high and low SNRs) held up. Four models were run in total evaluating the relationships shown in Fig. 1. In all cases, including age did not lead to a significant improvement in model fit compared to only including ITCP alone as a predictor. Age was never a significant main effect in these models (all *p* > 0.15).

### 3.2 Test-retest reliability

**Fig. 2.**
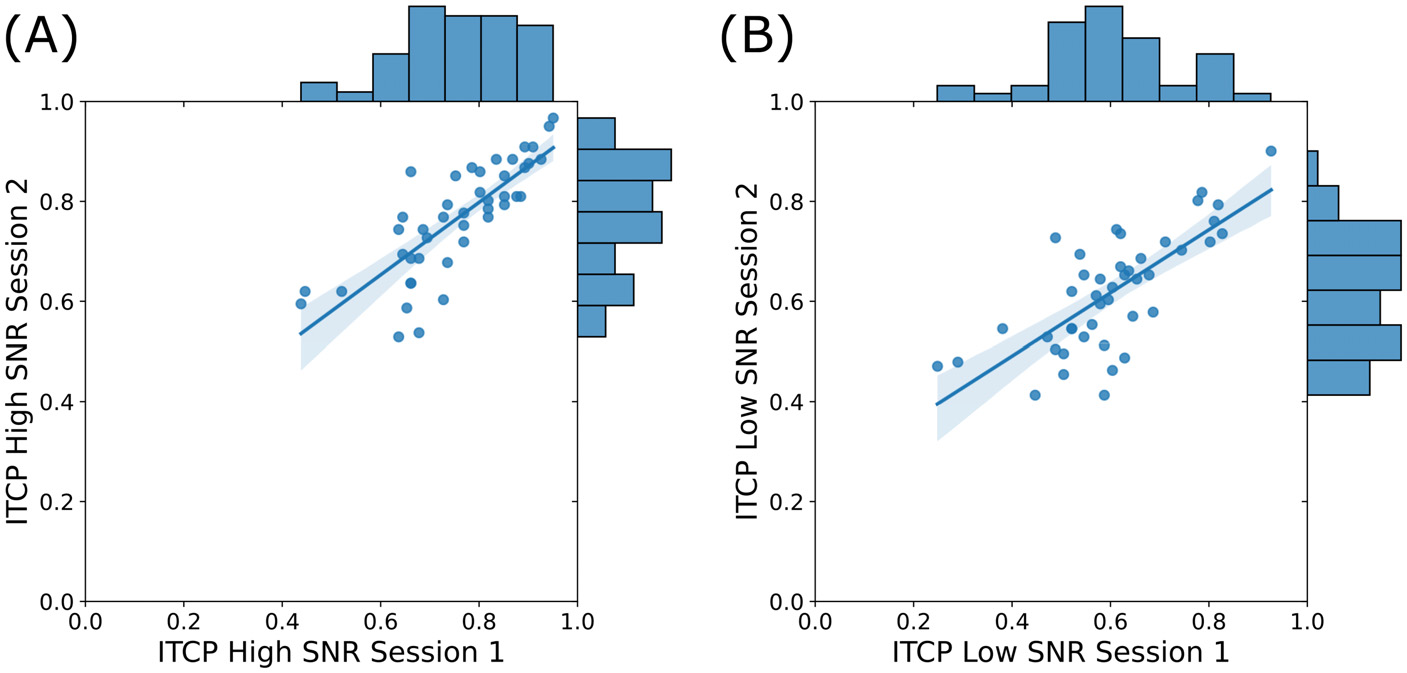
Scatterplots and histograms for ITCP test-retest reliability under both high- and low-SNR conditions. (A) Scatterplot of the high SNR ITCP from session 1 and session 2 along with distribution of the data (histograms). (B) Scatterplot of the low-SNR ITCP from session 1 and session 2 along with distribution of the data (histograms).

To assess the test-retest reliability of the ITCP, we utilized a subset of CI participants who participated in the ITCP two times on separate visits (with at least one year of device experience at each visit). Among these 44 participants who were measured twice, we calculated an estimate of reliability using the intraclass correlation coefficient (ICC) (Koo and Li, 2016) using the irr package in r (version 0.84.1) (Gamer *et al.*, 2019). Using this metric allowed us to assess absolute agreement between measurements at the first and second visits rather than just the predictability of one score from the other. We calculated this agreement separately for the high- and low-SNR conditions. We used an average-score, two-way random effects model of absolute agreement and found good agreement within the high-SNR condition across the two sessions, [ICC(*A*,2) = 0.887, *F*(43,44) = 8.82, *p* < 0.001] [Fig. 2(A)] and good agreement within the low-SNR condition across the two sessions [ICC(*A*,2) = 0.843, *F*(43,43.9) = 6.43, *p* < 0.001] [Fig. 2(B)]. To verify that there was not a significant learning effect between the first and second sessions of the ITCP, we compared mean accuracies at both time points for high- and low-SNR conditions. For the high-SNR condition, accuracies at visit 1 (*M* = 0.76, SD = 0.12) and visit 2 (*M* = 0.77, SD = 0.11) did not significantly differ [*t*(43) = −0.92, *p* = 0.358]. For the low-SNR condition, accuracies at visit 1 (*M* = 0.60, SD = 0.14) and visit 2 (M = 0.62, SD = 0.12) did not significantly differ [*t*(43) = −1.18, *p* = 0.244]. To evaluate the effect of the amount of time between session 1 and session 2, we ran a multiple regression predicting participants' second session scores from both their first session scores and the number of days between session 1 and session 2. For both the high-SNR and low-SNR conditions, the number of days between sessions did not reach significance [*t*(43) = −0.292, *p* = 0.771 and *t*(43) = −0.967, *p* = 0.339, respectively].

## 4. Discussion

Our results show the ITCP continues to show high convergent validity with common clinical speech recognition tasks and high test-retest reliability in CI users. Our sample included both pre- and post-lingually deafened CI users with a wide variety of device configurations. The fact that we still find good convergent validity is encouraging—participants who did well in our ITCP task tended to do well in common clinical measures. This shared variance between the ITCP and clinical measures is likely due to the fact that all three tasks are reliant on successful speech perception to respond accurately. The high test-retest reliability under both high- and low-SNR conditions is noteworthy given the large range of difference in times between data collection sessions. The high-SNR condition showed higher test-retest reliability than the low-SNR condition, which might reflect the fact that some CI users may still be learning to adapt to more challenging speech-in-noise scenarios even if their performance in more ideal speech recognition scenarios has stabilized.

The ITCP has multiple advantages in assessing CI users' speech recognition abilities and can be used to track improvements over time. While closed set single-word tasks are not currently favored in evaluating hearing outcomes [e.g., Yu and Schlauch (2019) and Zeitler *et al.* (2024)], we feel that the results presented here make a strong case for the use of the ITCP in both experimental research and clinical evaluation. The relatively balanced nature of both target words and their competitor response options gives the ITCP good diagnostic strength when evaluating speech perception abilities. Combine this with the ease of administration and scoring for the task, and it provides a unique opportunity to assess a listener's global speech perception abilities as well as follow-up and look for specific phonemic contrasts that prove challenging for the listener. Current experimental scripts could be expanded to include summary scores for each initial consonant or various phoneme categories. This could help guide clinicians when deciding on adjustments to CI users' device programming or inform decisions about how to best help a patient train their auditory system to process their new inputs (Glennon *et al.*, 2020). Critically, from the perspective of feasibility, the ITCP can also be run quickly. Our task that repeated every target word twice was about 15 min long.

While the timing of our presentation of auditory stimuli and response options did not allow us to conduct a response time analyses, another advantage of the ITCP is that response times can be collected and analyzed. There may be patients who perform well on the task in terms of accuracy but still self-report difficulties with speech in challenging listening scenarios—within the framework of the speed-accuracy trade-off, this may be reflected in longer response times (Heitz, 2014; Vaden *et al.*, 2022). Future work may investigate this more directly by incorporating ITCP materials into pupillometric or dual-task measures of listening effort.

In both NH listeners and CI patients, the ITCP can be presented at various noise levels to avoid ceiling effects [a common concern with closed set tasks; see Sommers *et al.* (1997)] even when only four response options are available. With the ITCP, we show strong convergent validity with two commonly accepted open set clinical tasks, which suggests that, despite its closed set nature, the task is still tapping into some shared speech perception processes that underlie all these tasks. However, it does so without the heavy demands on working memory, language processing, and speech production of open set sentence tasks, demands that may be problematic when testing children, people with mild cognitive impairment, or people with other atypical profiles. Therefore, regardless of the intuitive appeal of an open set task, properly designed closed set tasks may function just as well to assess speech perception abilities.

Using the ITCP, future studies could investigate if certain device configurations or other demographic factors can be used to predict what types of errors listeners are likely to make. Indeed, recent studies of CI users have highlighted various factors that are related to speech perception in noise by utilizing other tests, including duration of device use (Holder *et al.*, 2020), duration of deafness (Kitterick and Lucas, 2016), age (Berger *et al.*, 2023; for AzBio only), spectral and temporal resolution (Aldag and Nogueira, 2024; Choi *et al.*, 2023), and auditory cortical responses (Aldag and Nogueira, 2024; Berger *et al.*, 2023), although as indicated in the Introduction, some of these factors may be confounded by the specific cognitive requirements of particular tests. The closed set nature of the task also lends itself to being used for electroencephalogram (EEG) and/or pupillometry studies, as participants will not be preparing a motor response as they hear the word and response options can be withheld until after the onset of the auditory stimulus. This will allow for isolating auditory processes and avoiding the potential confound of preparatory motor responses [e.g., Zagha *et al.* (2022)].

## Supplementary Material

See the supplementary material for full demographic details as well as ITCP, CNC, and AzBio scores for each participant.

## Data Availability

The data that support the findings of this study are available within the article and its supplementary material.
